# Spontaneous sub-amputation from atypical critical limb ischemia in a patient without classical risk factors

**DOI:** 10.1016/j.idcr.2025.e02368

**Published:** 2025-09-13

**Authors:** Alsadig Suliman, Shorouq Mohammed Ali, Mohamed Saadeldein, Alkhansa Alkhider, Abubakr Muhammed

**Affiliations:** a Department of General Surgery, Sudan Medical Specialization Board, Khartoum, Khartoum, Sudan; b Department of General Surgery, Wad Medani University of Sciences and Technology, Wad Madani, Sudan; c Assistant professor of surgery, Department of General Surgery, University of Gezira, Wad Madni, Sudan; d Department of General Surgery, Gezira University Faculty of Medicine, Wad Madani, Sudan

**Keywords:** Case report, Spontaneous sub-amputation, Critical limb ischemia, Vascular access challenges, Peripheral arterial disease, Above-knee amputation (AKA)

## Abstract

**Background:**

Spontaneous sub-amputation is a rare manifestation of critical limb ischemia (CLI), most often associated with advanced vascular disease and comorbidities. We present a case of spontaneous foot sub-amputation in an elderly patient without major lifestyle-related risk factors, managed in a resource-limited setting.

**Case Report:**

A 76-year-old male with no history of diabetes, hypertension, or smoking presented after his left foot detached spontaneously during sleep, following two months of leg pain and two days of worsening discoloration and fever. On examination, he was septic, with absent left femoral and popliteal pulses and advanced gangrene. Imaging was not performed due to systemic and facility constraints. Emergency above-knee amputation was performed. Intraoperatively, thrombus removal was achieved using a Foley catheter in place of a Fogarty catheter. Postoperatively, the patient stabilized and showed good wound healing. Further diagnostics, including histopathology and thrombophilia screening, were not feasible due to financial and systemic limitations.

**Discussion:**

This case highlights an atypical CLI presentation in a patient without conventional lifestyle-associated risk factors, but with overlooked contributors such as prediabetes. The absence of imaging and vascular tools required improvisation for timely limb removal. The use of a Foley catheter for thrombectomy demonstrates adaptable practice in low-resource environments. Although the underlying etiology could not be fully confirmed, acute-on-chronic arterial occlusion was the most plausible diagnosis.

**Conclusion:**

Early recognition, sound clinical judgment, and surgical adaptability are vital in underserved settings. This case demonstrates that even with limited diagnostics and resources, effective outcomes can still be achieved.

## Highlights

• Rare spontaneous sub-amputation occurred without typical vascular risk factors.
• Atypical critical limb ischemia presented without traditional vascular risk factors.
• Improvised Foley catheter thrombectomy enabled limb-saving surgery in a tool-limited setting.
• Demonstrates surgical innovation and resilience in extreme resource-constrained environments.

## Introduction

Spontaneous sub-amputation refers to the natural detachment of a body part due to severe ischemia or infection, most often involving the distal toes or fingers [1], [2]. Proximal involvement, such as loss of an entire foot, is exceedingly rare and typically reflects advanced chronic ischemia, often compounded by overlooked risk factors [3]. Multiple causes—including diabetes, atherosclerosis, ainhum, pseudoainhum, malignancy-associated vascular compromise, and various gangrene types—can lead to this process [1], [2]. We describe a case of spontaneous foot sub-amputation in an elderly patient with no history of diabetes, hypertension, or smoking, but with contributory prediabetes, emphasizing the importance of early recognition and adaptive surgical intervention in atypical presentations.

## Case report

### Presentation & history

A 76-year-old retired male presented after noticing that his left foot had detached spontaneously during sleep, which he discovered upon waking to use the toilet. This event followed two months of exertional leg pain relieved by rest and two days of worsening symptoms, including foot blackening, sensory loss, weakness, and fever, without nausea, anorexia, or weight loss. Notably, he had no history of hypertension, diabetes, ischemic heart disease, or cerebrovascular events, was a non-smoker, and had no family history of vascular disease or hyperlipidemia. Given the patient’s advanced age and extent of tissue necrosis, the possibility of elder neglect was considered. However, the patient lived with attentive family members who provided consistent care and promptly sought medical attention upon symptom worsening. There were no signs of systemic neglect or abuse on clinical evaluation.

### Examination findings

**Fig. 1:**
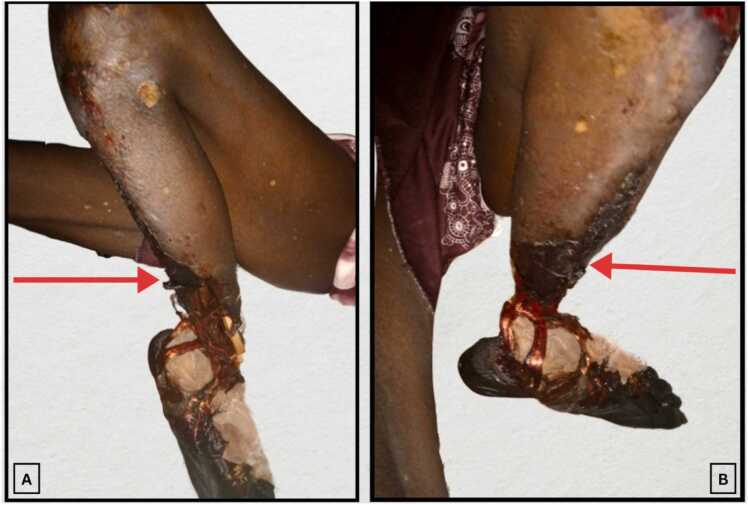
Clinical presentation of spontaneous sub-amputation. (A) Lateral and (B) medial views of the left lower limb showing extensive gangrene extending to the proximal calf and the natural plane of separation approximately 8 cm above the ankle, with visible bone transection and residual muscle attachments. An arrow indicates the plane of extent of gangrene.

On examination, he appeared acutely ill with a pulse of 125 bpm, respiratory rate of 33 cpm, blood pressure of 100/60 mmHg, temperature of 38.1°C, and a foul odor. The right lower limb was intact with hair loss but had palpable femoral, popliteal, and dorsalis pedis pulses, and an Ankle-Brachial Index (ABI) of 0.98. In contrast, the left lower limb was fixed in flexion at the hip and knee, with spontaneous sub-amputation 8 cm above the ankle; the foot remained attached by three muscle tendons while the bone was completely severed Fig. 1. The fixed flexion posture at the hip and knee was likely due to prolonged disuse and pain over the preceding weeks, rather than rapid contracture within two days. The patient’s limited mobility and chronic ischemic discomfort may have contributed to joint stiffness prior to acute deterioration. The affected limb showed extensive black discoloration up to the knee, significant calf tenderness, absent femoral and popliteal pulses, and an unmeasurable ABI. On the left side, posterior tibial, and dorsalis pedis pulses were weekly detected, indicating arterial pathology.

### Diagnostic workup and limitations

Laboratory findings shown in Table 1 indicated sepsis, leading to a clinical diagnosis of atherosclerotic dry gangrene with a superimposed infection that progressed to moist gangrene and spontaneous amputation. Upon initial evaluation, advanced vascular imaging such as Doppler ultrasound or CT angiography (CTA) was not performed due to a combination of clinical and systemic limitations. The patient arrived in the late evening in a state of acute sepsis and hemodynamic instability, requiring urgent resuscitation. Moreover, the nearest functional vascular imaging unit was located over an hour away and operated only during daytime hours, making timely transfer infeasible. A formal differential diagnosis was pursued postoperatively, as the patient was critically ill at presentation and required immediate surgical intervention. Laboratory investigations revealed leukocytosis (WBC: 18,000/μL), elevated CRP (110 mg/L) and significantly raised D-dimer (2.8 µg/mL), consistent with systemic inflammation and possible thromboembolic activity. Coagulation profile showed mild derangement (PT: 17.5 sec, aPTT: 43 sec, INR: 1.6). Serum creatinine was 2.8 mg/dL, most consistent with acute kidney injury from sepsis, as chronic kidney disease was excluded by normal renal size on ultrasound. HbA1c was 6.2 %, indicating prediabetes, but not sufficient to explain the severity of vascular disease. Syphilis serology was negative. ECG showed sinus tachycardia and chest X-ray was unremarkable. Histopathological examination or vascular biopsy—which might have confirmed underlying vasculitis, thromboembolism, or advanced arteriopathy—was also not performed. Although technically feasible, institutional policy required out-of-pocket payment for such analyses, and the patient was unable to afford the cost and declined the procedure. The absence of onsite, free pathology services further limited our diagnostic capacity. These constraints represent a notable limitation in establishing a definitive etiology. Nevertheless, based on the clinical presentation, intraoperative findings, and postoperative investigations, the most plausible diagnosis was critical limb ischemia due to acute-on-chronic peripheral arterial occlusion. Other differentials, including vasculitis, embolic phenomena, or hypercoagulable states, could not be excluded but were not confirmed due to systemic limitations.

**Table 1:** Laboratory and Imaging Investigations with Reference Ranges.

**Test**	**Result**	**Reference Range**
**White Blood Cell Count (WBC)**	18,000 /µL	4000 – 11,000 /µL
**Hemoglobin (Hb)**	9.5 g/dL	13.5 – 17.5 g/dL (male)
**Platelet Count**	250,000 /µL	150,000 – 400,000 /µL
**C-Reactive Protein (CRP)**	110 mg/L	< 5 mg/L
**D-dimer**	2.8 µg/mL	< 0.5 µg/mL
**Prothrombin Time (PT)**	17.5 s	11 – 15 s
**Activated Partial Thromboplastin Time (aPTT)**	43 s	25 – 35 s
**INR**	1.6	0.8 – 1.2
**Hemoglobin A1c (HbA1c)**	6.2 %	< 5.7 % (normal), 5.7–6.4 % (pre-diabetes)
**Random Blood Glucose**	100 mg/dL	70 – 140 mg/dL
**Creatinine**	2.8 mg/dL	0.6 – 1.3 mg/dL
**Serology for Syphilis**	Negative	Negative
**Chest X-ray**	Normal	–
**Electrocardiogram (ECG)**	Sinus tachycardia	Normal sinus rhythm
**Ultrasound Abdomen (after 1 week)**	No significant abnormality detected; kidneys normal in size	Normal

### Operative findings and management

**Fig. 2:**
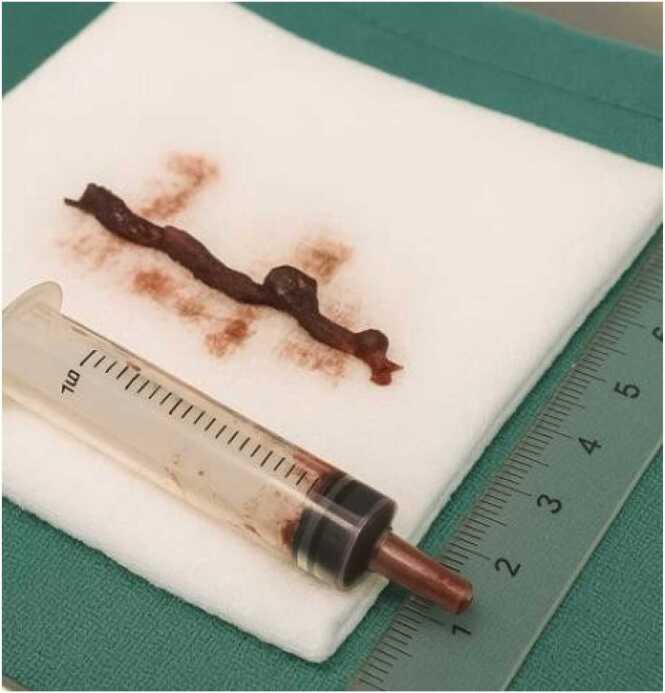
Extracted thrombus specimen obtained using a Foley catheter. Segment of thrombus removed intraoperatively via an improvised technique using a Foley urinary catheter, placed beside a 10 mL syringe and a measurement scale for size reference. The thrombus was extracted from the femoral artery following spontaneous sub-amputation and septic presentation.

Given the patient's unstable condition and the unavailability of emergent imaging, the decision was made to proceed directly to surgical intervention. Intraoperatively, no blood flow was detected in the femoral artery. Lacking a Fogarty catheter, a size 6 two-way Foley urinary catheter was used for thrombus extraction as shown in Fig. 2, by advancing it beyond the thrombus, inflating the balloon with sterile saline, and Withdrawing it slowly. After two attempts, this maneuver successfully removed the thrombus and restored blood flow. Intraoperatively, the femoral artery appeared thickened and rigid, consistent with chronic arteriosclerosis. The extracted thrombus was partially organized and laminated, supporting the diagnosis of acute-on-chronic arterial occlusion. Although an above-knee amputation was the primary intervention, proximal arterial exploration and thrombus extraction were performed to assess perfusion and ensure that a more proximal (hip disarticulation) amputation was not needed. Additionally, removal of septic thrombotic material may have contributed to systemic infection control. A video demonstrating intraoperative thrombus extraction using a Foley catheter is provided in Video 1.

Supplementary material related to this article can be found online at doi:10.1016/j.idcr.2025.e02368.

The following is the Supplementary material related to this article Video S1.

Video S1

### Postoperative course and follow-up

The patient remained hospitalized for five days postoperatively. During this period, he received intravenous broad-spectrum antibiotics, wound care, analgesia, and three units of packed red blood cells. His white blood cell counts normalized, and he became clinically stable. Blood pressure was closely monitored during hospitalization and follow-up, and remained within the low-to-normal range, with no evidence of occult hypertension. Echocardiography was performed to assess for potential cardiac sources of emboli and revealed no abnormalities. As atrial fibrillation may be paroxysmal, we considered extended outpatient cardiac rhythm monitoring (e.g., Holter or Zio Patch) to rule out occult embolic sources; however, these options were not available due to systemic and financial constraints in our setting. During his two-week follow-up, the surgical site showed appropriate healing with no signs of infection or wound breakdown. The patient reported improved appetite and energy levels, though he remained functionally dependent on mobility. By one month, the wound had fully healed, and no new ischaemic changes were noted in the contralateral limb. The patient had been referred for outpatient vascular surgery evaluation, including an assessment of the upper limbs to rule out systemic vascular involvement. Prosthetic rehabilitation planning was initiated in collaboration with a multidisciplinary team, and he started on long-term secondary prevention, including aspirin and statin therapy. Although formal thrombophilia testing and vascular imaging remained inaccessible, the patient was enrolled in regular follow-up for cardiovascular risk factor screening and surveillance for recurrence. He was also referred to social and psychological support to aid in adjustment to life post-amputation. At the time of the last review (six weeks post-surgery), the patient was stable, engaged in outpatient care and beginning assisted mobility training at home. A visual summary of the patient’s clinical course, including symptom onset, surgical intervention, and recovery milestones, is provided in Supplemental Figure 1.

## Discussion

### Rarity and definition

Spontaneous sub-amputation typically follows severe dry gangrene. Although rare, it has been reported in malignancies of the penis, appendix, breast, and tongue [2], [4], [5], [6], [7], [8], [9]. This case is notable for its rapid progression to sub-amputation without prior vascular intervention.

### Risk factors and contributing conditions

Unlike most cases linked to diabetes or smoking, this patient had no overt lifestyle risk factors but did have overlooked contributors such as prediabetes, raising the possibility of occult vascular pathology or hypercoagulability or genetic predisposition. A delayed diagnosis likely exacerbated ischemia, necessitating urgent surgical management.

### Management challenges in resource-limited settings

Revascularization was not attempted because the patient presented in septic shock with extensive gangrene, and our facility lacked vascular bypass capability, imaging, and Fogarty catheters. Attempting revascularization in such a setting could have risked severe reperfusion injury and worsening of systemic sepsis. Therefore, the decision for above-knee amputation was made promptly as a life-saving intervention. Moreover, the use of an improvised Foley catheter for thrombus extraction highlights the challenges in resource-limited settings where standard Fogarty catheters are unavailable [1], [10], [11]. In the absence of a Fogarty catheter, alternative tools such as a Freer elevator, vessel loop, or small suction catheter could be cautiously considered for thrombus extraction. However, these lack balloon mechanisms and may increase the risk of intimal injury or incomplete clot removal [10], [11]. Postoperative care included antibiotics, resuscitation, and blood transfusion. Secondary prevention was limited to aspirin and statin therapy, as anticoagulation and intensive monitoring were not feasible.

### Diagnostic considerations and limitations

A systematic diagnostic strategy is essential. It begins with a comprehensive clinical history to identify risk factors, comorbidities, and the evolution of symptoms. A focused physical examination—using tools such as ABI, Doppler ultrasonography, and skin perfusion assessment—evaluates limb viability, while advanced imaging (CTA or MRA) precisely delineates vascular occlusions and embolic sources. In resource-limited settings, bedside Doppler studies coupled with a detailed clinical evaluation are indispensable. Laboratory tests should include coagulation studies, inflammatory markers, lipid profiles, and autoimmune screening, with vascular biopsy serving as the confirmatory test for vasculitis. Early multidisciplinary consultation, particularly with vascular surgery, is crucial to prevent further ischemic injury [10].

### Comparisons with literature

Literature shows that spontaneous sub-amputation in non-diabetic, non-hypertensive individuals is extremely rare [12], [13]. Similar cases, such as one reported by Mani et al. in a diabetic patient, emphasize that early signs like exertional leg pain should prompt timely vascular screening to prevent limb loss [1]. Unlike tropical idiopathic gangrene, this case suggests a severe but undiagnosed form of peripheral arterial disease [3].

### Long-Term Care and Outcomes

Long-term care requires secondary prevention strategies (antiplatelet therapy, statins, vascular follow-up), further evaluation for hypercoagulability, and comprehensive rehabilitation— including prosthetic fitting and mental health support—to address the significant psychosocial and functional impacts of amputation [14], [15]. The patient was followed up at two weeks and one-month post-discharge. Clinical evaluation confirmed wound healing without complications or ischemic changes in the contralateral limb. Although vascular biopsy and thrombophilia screening were unavailable, outpatient referral to a tertiary vascular center was arranged for further systemic evaluation. Despite the diagnostic limitations, the patient achieved satisfactory short-term outcomes through timely intervention and adaptive postoperative care.

## Conclusion

This case demonstrates that spontaneous sub-amputation can occur even without overt vascular risk factors, with prediabetes status emerging as overlooked contributor. It highlights the necessity of maintaining high suspicion for critical limb ischemia in atypical presentations. In resource-limited settings, adaptive surgical strategies—such as improvising with a Foley catheter—reinforce how clinical flexibility can determine survival when standard tools are unavailable.

## Ethical approval

This case report did not require ethics approval, as it involves a single patient and does not constitute research according to the institution's guidelines.

## Funding

No extramural funds were used to support this case report.

## CRediT authorship contribution statement

**Abubakr Muhammed:** Visualization, Software, Formal analysis. **Alkhansa Alkhider:** Writing – review & editing, Resources, Investigation, Data curation. **Mohamed Saadeldein:** Writing – review & editing, Supervision, Conceptualization. **Shorouq Mohammed Ali:** Writing – original draft, Validation, Investigation, Data curation, Conceptualization. **Alsadig Suliman:** Writing – review & editing, Writing – original draft, Visualization, Validation, Supervision, Software, Resources, Project administration, Methodology, Investigation, Funding acquisition, Formal analysis, Data curation, Conceptualization.

## Authorship Statement

All authors have made substantial contributions to one or more of the following aspects of the work:

• The conception and design of the study, acquisition of data, or analysis and interpretation of data;
• Drafting the manuscript or revising it critically for important intellectual content;
• Final approval of the version to be submitted.

We confirm that Dr. Alsadig Suliman has been designated as the corresponding author and will be responsible for all communication with the editorial office during the submission and review process. All authors agree to be accountable for all aspects of the work to ensure that any questions related to the accuracy or integrity of any part of the work are appropriately investigated and resolved.

## Submission Declaration and Authorship Statement

We hereby declare that the work described in our submitted manuscript has not been published previously, except in the form of a preprint, an abstract, a published lecture, academic thesis, or registered report, as outlined in Elsevier’s policy on multiple, redundant, or concurrent publication.

The manuscript is not under consideration for publication elsewhere.

All authors have approved the final version of the manuscript and, where applicable, the responsible authorities at the institution(s) where the work was carried out have granted their approval for submission.

If accepted, this article will not be published elsewhere in the same form, in English or in any other language, including electronically, without the written consent of the copyright holder.

We acknowledge that the journal may use plagiarism detection software or other screening tools to verify compliance with publishing policies.

## Declaration of Generative AI and AI-assisted technologies in the writing process

During the preparation of this manuscript, the authors used OpenAI's ChatGPT (GPT-) to assist with language refinement, grammar correction, and improvement of readability. The AI was not used for clinical content creation, data interpretation, or scientific analysis. All content was reviewed and edited by the authors, who take full responsibility for the integrity and accuracy of the manuscript.

## Declaration of Competing Interest

The authors declare that they have no known competing financial interests or personal relationships that could have appeared to influence the work reported in this paper.

## Footnotes

Appendix A Supplementary data associated with this article can be found in the online version at doi:10.1016/j.idcr.2025.e02368.
