# Supplementary material for: Spontaneous sub-amputation from atypical critical limb ischemia in a patient without classical risk factors
Source: IDCases. 2025 Sep 13;42:e02368. doi: 10.1016/j.idcr.2025.e02368 (PMC12466145; doi:10.1016/j.idcr.2025.e02368)
Supplement: Supplementary file 2 — Supplementary material [file mmc1.docx]

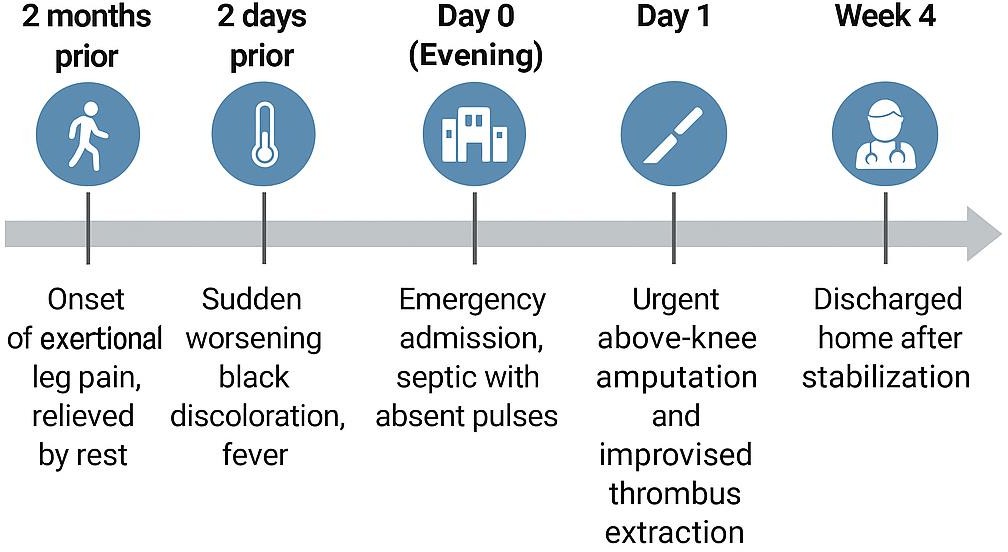


**Supplemental Figure 1: Clinical timeline of disease progression and intervention.**

Visual summary of the patient's clinical course: from initial onset of exertional leg pain two months prior, to spontaneous sub-amputation and septic presentation on Day 0. Urgent

above-knee amputation with improvised thrombus extraction was performed on Day 1. The

patient stabilized postoperatively and was discharged home by Week 4. This figure highlights the accelerated progression and surgical decision points in a resource-limited setting.
